# Supplementary material for: Monitoring Carbapenem-Resistant Enterobacterales in the Environment to Assess the Spread in the Community
Source: Antibiotics (Basel). 2022 Jul 8;11(7):917. doi: 10.3390/antibiotics11070917 (PMC9311640; doi:10.3390/antibiotics11070917)
Supplement: Supplementary file 1 [file antibiotics-11-00917-s001.zip › antibiotics-1782633-supplementary.pdf]

Supplementary Materials

to Monitoring Carbapenem-Resistant Enterobacterales in the Environment to Assess the Spread in the Community by Taro Uras, Saki Goto and Mio Sato.

Table S1. Detailed information (sampling locations, carbapenem-resistant genes, isolation conditions, and accession numbers) of carbapenem-resistant isolates obtained in this study is shown in this table. The assembled sequences obtained by the primers 518F and 800R were registered for the isolates 1-146 and 148-223, while the sequences obtained by the primer 800R were registered for the isolates 147 and 224-247.

No.	Sampling dates	Location	Genus	Gene encoding carbapenemase	Incubation method	accession no.
1_5_9	13-Nov-2018	A2	*Enterobacter*	Not Detected	42°C W/O Zn	ON845217
2_42_10	27-Nov-2018	B7*	*Klebsiella*	*bla* $_{GES}$ (CP)	42°C W/O Zn	ON845218
3_e8	20-Aug-2019	A1	*Klebsiella*	*bla* $_{GES}$ (CP)	42°C W/O Zn	ON845219
4_e5	20-Aug-2019	A1	*Pseudomonas****	Not Detected	42°C W/O Zn	**
5_e4	20-Aug-2019	A1	*Klebsiella*	*bla* $_{GES}$ (CP)	42°C W/O Zn	ON845220
6_d8	20-Aug-2019	A1	*Klebsiella*	*bla* $_{GES}$ (CP)	42°C W/O Zn	ON845221
7_d5	20-Aug-2019	A1	*Enterobacter*	*bla* $_{IMP-1}$	42°C W/O Zn	ON845222
8_d4	20-Aug-2019	A1	*Klebsiella*	*bla* $_{GES}$ (CP)	42°C W/O Zn	ON845223
9_c7	20-Aug-2019	A1	*Klebsiella*	Not Detected	42°C W/O Zn	ON845224
10_c3	20-Aug-2019	A1	*Pseudomonas*		42°C W/O Zn	ON845225
11_c6	20-Aug-2019	A1	*Cupriavidus*		42°C W/O Zn	ON845226
12_b3	20-Aug-2019	A1	*Pseudomonas*	Not Detected	42°C W/O Zn	ON845227
13_s4_61	24-Sep-2019	TR	*Klebsiella*	*bla* $_{GES}$ (CP)	42°C W/O Zn	ON845228
14_s5_62	24-Sep-2019	TR	*Pseudomonas*		42°C W/O Zn	ON845229
15_s6_67	24-Sep-2019	TR	*Stenotrophomonas*		42°C W/O Zn	ON845230
16_s7_71	24-Sep-2019	TR	*Pseudomonas*		42°C W/O Zn	ON845231
17_37_k7_1	28-Oct-2019	K7	*Stenotrophomonas*		37°C W/O Zn	ON845232
18_37_k11_60	28-Oct-2019	K11	*Aeromonas*		37°C W/O Zn	ON845233
19_37_k11_11	28-Oct-2019	K11	*Stenotrophomonas*		37°C W/O Zn	ON845234
20_37_k11_13	28-Oct-2019	K11	*Stenotrophomonas*		37°C W/O Zn	ON845235
21_37_k11_18	28-Oct-2019	K11	*Stenotrophomonas*		37°C W/O Zn	ON845236
22_37_k11_19	28-Oct-2019	K11	*Pseudomonas*		37°C W/O Zn	ON845237
23_37_k11_41	28-Oct-2019	K11	*Stenotrophomonas*		37°C W/O Zn	ON845238
24_37_k11_61	28-Oct-2019	K11	*Stenotrophomonas*		37°C W/O Zn	ON845239
25_37_k11_10	28-Oct-2019	K11	*Pseudomonas****		37°C W/O Zn	ON845240

26_37_k11_33	28-Oct-2019	K11	*Stenotrophomonas*		37°C W/O Zn	ON845241
27_42_k7_2	28-Oct-2019	K7	*Pseudomonas*		42°C W/O Zn	ON845242
28_42_k7_3	28-Oct-2019	K7	*Pseudomonas*		42°C W/O Zn	ON845243
29_h_37_1	25-Nov-2019	A1	*Enterobacter*	*bla* GES (CP)	37°C W/O Zn	ON845244
30_h_37_5	25-Nov-2019	A1	*Enterobacter*	*bla* GES (CP)	37°C W/O Zn	ON845245
31_h_37_8	25-Nov-2019	A1	*Enterobacter*	*bla* GES (CP)	37°C W/O Zn	ON845246
32_h_37_13	25-Nov-2019	A1	*Enterobacter*	*bla* GES (CP)	37°C W/O Zn	ON845247
33_h_37_20	25-Nov-2019	A1	*Enterobacter*	*bla* GES (CP)	37°C W/O Zn	ON845248
34_h_37_40	25-Nov-2019	A1	*Enterobacter*	*bla* GES (CP)	37°C W/O Zn	ON845249
35_h_42_2	25-Nov-2019	A1	*Enterobacter*	*bla* GES (CP)	42°C W/O Zn	ON845250
36_h_42_3	25-Nov-2019	A1	*Klebsiella*	*bla* GES (CP)	42°C W/O Zn	ON845251
37_h_42_5	25-Nov-2019	A1	*Enterobacter*	*bla* GES (CP)	42°C W/O Zn	ON845252
38_h_42_9	25-Nov-2019	A1	*Enterobacter*	*bla* GES (ESBL)	42°C W/O Zn	ON845253
39_h_42_12	25-Nov-2019	A1	*Klebsiella*	Not Detected	42°C W/O Zn	ON845254
40_h_42_17	25-Nov-2019	A1	*Enterobacter*	Not Detected	42°C W/O Zn	ON845255
41_h_42_20	25-Nov-2019	A1	*Enterobacter*	*bla* GES (CP)	42°C W/O Zn	ON845256
42_h_42_25	25-Nov-2019	A1	*Enterobacter*	*bla* GES (CP)	42°C W/O Zn	ON845257
43_h_42_27	25-Nov-2019	A1	*Enterobacter*	*bla* GES (CP)	42°C W/O Zn	ON845258
44_h_42_29	25-Nov-2019	A1	*Enterobacter*	*bla* GES (ESBL)	42°C W/O Zn	ON845259
45_h_42_32	25-Nov-2019	A1	*Enterobacter*	*bla* GES (ESBL)	42°C W/O Zn	ON845260
46_h_42_33	25-Nov-2019	A1	*Enterobacter*	*bla* GES (ESBL)	42°C W/O Zn	ON845261
47_h_42_37	25-Nov-2019	A1	*Enterobacter*	Not Detected	42°C W/O Zn	ON845262
48_h_4_38	25-Nov-2019	A1	*Enterobacter*	*bla* GES (ESBL)	42°C W/O Zn	ON845263
49_h4_2_39	25-Nov-2019	A1	*Enterobacter*	*bla* GES (ESBL)	42°C W/O Zn	ON845264
50_h_42_40	25-Nov-2019	A1	*Enterobacter*	*bla* GES (ESBL)	42°C W/O Zn	ON845265
51_h_42_42	25-Nov-2019	A1	*Enterobacter*	*bla* GES (ESBL)	42°C W/O Zn	ON845266
52_h_42_45	25-Nov-2019	A1	*Enterobacter*	*bla* GES (ESBL)	42°C W/O Zn	ON845267
53_h_42_47	25-Nov-2019	A1	*Enterobacter*	*bla* GES (CP)	42°C W/O Zn	ON845268
54_h_42_48	25-Nov-2019	A1	*Enterobacter*	*bla* GES (ESBL)	42°C W/O Zn	ON845269
55_h_42_52	25-Nov-2019	A1	*Enterobacter*	*bla* GES (CP)	42°C W/O Zn	ON845270
56_h_42_59	25-Nov-2019	A1	*Enterobacter*	*bla* GES (CP)	42°C W/O Zn	ON845271
57_h_42_59	25-Nov-2019	A1	*Shigella*	*bla* GES (CP)	42°C W/O Zn	ON845272
58_h_42_62	25-Nov-2019	A1	*Shigella*	Not Detected	42°C W/O Zn	ON845273
59_h_42_64	25-Nov-2019	A1	*Shigella*	Not Detected	42°C W/O Zn	ON845274

60_t_37_2	25-Nov-2019	A2	*Enterobacter*	*bla* GES (CP)	37°C W/O Zn	ON845275
61_37_1	6-Jul-2020	A2	*Stenotrophomonas*		37°C W/O Zn	ON845276
62_37_2	6-Jul-2020	A2	*Stenotrophomonas*		37°C W/O Zn	ON845277
63_37_4	6-Jul-2020	A2	*Stenotrophomonas*		37°C W/O Zn	ON845278
64_37_8	6-Jul-2020	A2	*Stenotrophomonas*		37°C W/O Zn	ON845279
65_37_9	6-Jul-2020	A2	*Stenotrophomonas*		37°C W/O Zn	ON845280
66_37_13	6-Jul-2020	A2	*Stenotrophomonas*		37°C W/O Zn	ON845281
67_37_14	6-Jul-2020	A2	*Stenotrophomonas*		37°C W/O Zn	ON845282
68_37_15	6-Jul-2020	A2	*Stenotrophomonas*		37°C W/O Zn	ON845283
69_37_16	6-Jul-2020	A2	*Stenotrophomonas*		37°C W/O Zn	ON845284
70_37_18	6-Jul-2020	A2	*Stenotrophomonas*		37°C W/O Zn	ON845285
71_37_20	6-Jul-2020	A2	*Stenotrophomonas*		37°C W/O Zn	ON845286
72_37_21	6-Jul-2020	A2	*Stenotrophomonas*		37°C W/O Zn	ON845287
73_37_22	6-Jul-2020	A2	*Stenotrophomonas*		37°C W/O Zn	ON845288
74_37_24	6-Jul-2020	A2	*Pseudomonas****		37°C W/O Zn	ON845289
75_37_25	6-Jul-2020	A2	*Stenotrophomonas*		37°C W/O Zn	ON845290
76_37_26	6-Jul-2020	A2	*Stenotrophomonas*		37°C W/O Zn	ON845291
77_37_27	6-Jul-2020	A2	*Pseudomonas****		37°C W/O Zn	ON845292
78_37_28	6-Jul-2020	A2	*Stenotrophomonas*		37°C W/O Zn	ON845293
79_37_29	6-Jul-2020	A2	*Stenotrophomonas*		37°C W/O Zn	ON845294
80_37_29	6-Jul-2020	A2	*Pseudomonas****		37°C W/O Zn	ON845295
81_37_35	6-Jul-2020	A2	*Stenotrophomonas*		37°C W/O Zn	ON845296
82_37_36	6-Jul-2020	A2	*Stenotrophomonas*		37°C W/O Zn	ON845297
83_37_39	6-Jul-2020	A2	*Stenotrophomonas*		37°C W/O Zn	ON845298
84_37_40	6-Jul-2020	A2	*Stenotrophomonas*		37°C W/O Zn	ON845299
85_37_42	6-Jul-2020	A2	*Pseudomonas****		37°C W/O Zn	ON845300
86_37_44	6-Jul-2020	A2	*Stenotrophomonas*		37°C W/O Zn	ON845301
87_37_45	6-Jul-2020	A2	*Stenotrophomonas*		37°C W/O Zn	ON845302
88_37_52	6-Jul-2020	A2	*Stenotrophomonas*		37°C W/O Zn	ON845303
89_37_54	6-Jul-2020	A2	*Stenotrophomonas*		37°C W/O Zn	ON845304
90_42_3	6-Jul-2020	A2	*Pseudomonas****		42°C W/O Zn	ON845305
91_42_5	6-Jul-2020	A2	*Pseudomonas****		42°C W/O Zn	ON845306
92_42_6	6-Jul-2020	A2	*Pseudomonas****		42°C W/O Zn	ON845307
93_42_9	6-Jul-2020	A2	*Pseudomonas****		42°C W/O Zn	ON845308

94_42_10	6-Jul-2020	A2	*Stenotrophomonas*		42°C W/O Zn	ON845309
95_42_11	6-Jul-2020	A2	*Stenotrophomonas*		42°C W/O Zn	ON845310
96_42_16	6-Jul-2020	A2	*Stenotrophomonas*		42°C W/O Zn	ON845311
97_42_20	6-Jul-2020	A2	*Stenotrophomonas*		42°C W/O Zn	ON845312
98_42_22	6-Jul-2020	A2	*Pseudomonas******		42°C W/O Zn	ON845313
99_42_23	6-Jul-2020	A2	*Stenotrophomonas*		42°C W/O Zn	ON845314
100_42_24	6-Jul-2020	A2	*Pseudomonas******		42°C W/O Zn	ON845315
101_42_27	6-Jul-2020	A2	*Stenotrophomonas*		42°C W/O Zn	ON845316
102_42_28	6-Jul-2020	A2	*Stenotrophomonas*		42°C W/O Zn	ON845317
103_g_37_2	26-Oct-2020	B1	*Stenotrophomonas*		37°C W/O Zn	ON845318
104_g_37_6	26-Oct-2020	B1	*Stenotrophomonas*		37°C W/O Zn	ON845319
105_g_37_13	26-Oct-2020	B1	*Escherichia*	*bla* IMP-1 + *bla* NDM	37°C W/O Zn	ON845320
106_g_37_14	26-Oct-2020	B1	*Escherichia*	*bla* IMP-1 + *bla* NDM	37°C W/O Zn	ON845321
107_g_37_16	26-Oct-2020	B1	*Escherichia*	*bla* NDM	37°C W/O Zn	ON845322
108_g_37_18	26-Oct-2020	B1	*Stenotrophomonas*		37°C W/O Zn	ON845323
109_g_37_19	26-Oct-2020	B1	*Stenotrophomonas*		37°C W/O Zn	ON845324
110_g_37_25	26-Oct-2020	B1	*Pseudomonas******		37°C W/O Zn	ON845325
111_g_37_25	26-Oct-2020	B1	*Stenotrophomonas*		37°C W/O Zn	ON845326
112_g_37_35	26-Oct-2020	B1	*Stenotrophomonas*		37°C W/O Zn	ON845327
113_g_37_36	26-Oct-2020	B1	*Stenotrophomonas*		37°C W/O Zn	ON845328
114_g_42_2	26-Oct-2020	B1	*Ralstonia*		42°C W/O Zn	ON845329
115_g_42_9	26-Oct-2020	B1	*Escherichia*	*bla* IMP-1 + *bla* NDM	42°C W/O Zn	ON845330
116_g_42_11	26-Oct-2020	B1	*Escherichia*	*bla* IMP-1 + *bla* NDM	42°C W/O Zn	ON845331
117_g_42_12	26-Oct-2020	B1	*Pseudomonas*		42°C W/O Zn	ON845332
118_g_42_14	26-Oct-2020	B1	*Cupriavidus*		42°C W/O Zn	ON845333
119_s_37_1	26-Oct-2020	B1	*Stenotrophomonas*		37°C W/O Zn	ON845334
120_s_37_2	26-Oct-2020	B1	*Stenotrophomonas*		37°C W/O Zn	ON845335
121_s_37_3	26-Oct-2020	B1	*Stenotrophomonas*		37°C W/O Zn	ON845336
122_s_37_4	26-Oct-2020	B1	*Stenotrophomonas*		37°C W/O Zn	ON845337
123_s_37_5	26-Oct-2020	B1	*Stenotrophomonas*		37°C W/O Zn	ON845338
124_s_37_6	26-Oct-2020	B1	*Stenotrophomonas*		37°C W/O Zn	ON845339
125_s_37_15	26-Oct-2020	B1	*Stenotrophomonas*		37°C W/O Zn	ON845340
126_s_37_17	26-Oct-2020	B1	*Stenotrophomonas*		37°C W/O Zn	ON845341
127_s_37_18	26-Oct-2020	B1	*Stenotrophomonas*		37°C W/O Zn	ON845342

128_s_37_19	26-Oct-2020	B1	*Stenotrophomonas*		37°C W/O Zn	ON845343
129_s_37_21	26-Oct-2020	B1	*Pseudomonas******		37°C W/O Zn	ON845344
130_s_37_23	26-Oct-2020	B1	*Pseudomonas******		37°C W/O Zn	ON845345
131_s_37_24	26-Oct-2020	B1	*Stenotrophomonas*		37°C W/O Zn	ON845346
132_s_37_29	26-Oct-2020	B1	*Stenotrophomonas*		37°C W/O Zn	ON845347
133_s_37_31	26-Oct-2020	B1	*Stenotrophomonas*		37°C W/O Zn	ON845348
134_37_3	19-Apr-2021	A1	*Raoultella*	*bla* GES (CP)	37°C W/O Zn	ON845349
135_37_5	19-Apr-2021	A1	*Enterobacter*	*bla* GES (CP)	37°C W/O Zn	ON845350
136_37_6	19-Apr-2021	A1	*Klebsiella*	*bla* GES (CP)	37°C W/O Zn	ON845351
137_37_7	19-Apr-2021	A1	*Enterobacter*	*bla* GES (CP)	37°C W/O Zn	ON845352
138_37_8	19-Apr-2021	A1	*Raoultella*	*bla* GES (CP)	37°C W/O Zn	ON845353
139_37_9	19-Apr-2021	A1	*Enterobacter*	*bla* GES (CP)	37°C W/O Zn	ON845354
140_37_10	19-Apr-2021	A1	*Enterobacter*	*bla* GES (CP)	37°C W/O Zn	ON845355
141_37_11	19-Apr-2021	A1	*Enterobacter*	Not Detected	37°C W/O Zn	ON845356
142_37_13	19-Apr-2021	A1	*Enterobacter*	*bla* GES (CP)	37°C W/O Zn	ON845357
143_37_15	19-Apr-2021	A1	*Klebsiella*	Not Detected	37°C W/O Zn	ON845358
144_37_16	19-Apr-2021	A1	*Enterobacter*	Not Detected	37°C W/O Zn	ON845359
145_37_17	19-Apr-2021	A1	*Klebsiella*	Not Detected	37°C W/O Zn	ON845360
146_37_18	19-Apr-2021	A1	*Raoultella*	*bla* GES (CP)	37°C W/O Zn	ON845361
147_37_21	19-Apr-2021	A1	*Klebsiella*	Not Detected	37°C W/O Zn	ON844997
148_37_23	19-Apr-2021	A1	*Enterobacter*	*bla* GES (CP)	37°C W/O Zn	ON845362
149_37_26	19-Apr-2021	A1	*Klebsiella*	Not Detected	37°C W/O Zn	ON845363
150_37_28	19-Apr-2021	A1	*Klebsiella*	Not Detected	37°C W/O Zn	ON845364
151_37_33	19-Apr-2021	A1	*Klebsiella*	*bla* GES (CP)	37°C W/O Zn	ON845365
152_37_35	19-Apr-2021	A1	*Enterobacter*	Not Detected	37°C W/O Zn	ON845366
153_37_36	19-Apr-2021	A1	*Klebsiella*	Not Detected	37°C W/O Zn	ON845367
154_37_37	19-Apr-2021	A1	*Klebsiella*	*bla* GES (CP)	37°C W/O Zn	ON845368
155_42_1	19-Apr-2021	A1	*Klebsiella*	*bla* IMP-1	42°C W/O Zn	ON845369
156_42_3	19-Apr-2021	A1	*Klebsiella*	Not Detected	42°C W/O Zn	ON845370
157_42_4	19-Apr-2021	A1	*Klebsiella*	Not Detected	42°C W/O Zn	ON845371
158_42_5	19-Apr-2021	A1	*Klebsiella*	Not Detected	42°C W/O Zn	ON845372
159_42_6	19-Apr-2021	A1	*Klebsiella*	Not Detected	42°C W/O Zn	ON845373
160_42_8	19-Apr-2021	A1	*Enterobacter*	*bla* GES (CP)	42°C W/O Zn	ON845374
161_1_37_1	21-Jun-2021	A3	*Escherichia*	*bla* NDM	37°C W/O Zn	ON845375

162_1_37_2	21-Jun-2021	A3	*Aeromonas*		37°C W/O Zn	ON845376
163_1_37_7	21-Jun-2021	A3	*Aeromonas*		37°C W/O Zn	ON845377
164_1_37_9	21-Jun-2021	A3	*Raoultella*	*bla* $_{GES}$ (CP)	37°C W/O Zn	ON845378
165_1_37_12	21-Jun-2021	A3	*Aeromonas*		37°C W/O Zn	ON845379
166_1_37_13	21-Jun-2021	A3	*Enterobacter*	*bla* $_{GES}$ (CP)	37°C W/O Zn	ON845380
167_1_37_14	21-Jun-2021	A3	*Stenotrophomonas*		37°C W/O Zn	ON845381
168_1_37_16	21-Jun-2021	A3	*Aeromonas*		37°C W/O Zn	ON845382
169_1_37_19	21-Jun-2021	A3	*Aeromonas*		37°C W/O Zn	ON845383
170_1_42_1	21-Jun-2021	A3	*Enterobacter*	Not Detected	42°C W/O Zn	ON845384
171_1_42_5	21-Jun-2021	A3	*Klebsiella*	*bla* $_{GES}$ (CP)	42°C W/O Zn	ON845385
172_1_42_7	21-Jun-2021	A3	*Citrobacter*	*bla* $_{GES}$ (CP)	42°C W/O Zn	ON845386
173_1_42_12	21-Jun-2021	A3	*Enterobacter*	*bla* $_{GES}$ (CP)	42°C W/O Zn	ON845387
174_1_42_14	21-Jun-2021	A3	*Raoultella*	*bla* $_{GES}$ (CP)	42°C W/O Zn	ON845388
175_1_42_15	21-Jun-2021	A3	*Enterobacter*	*bla* $_{GES}$ (CP)	42°C W/O Zn	ON845389
176_1_42_16	21-Jun-2021	A3	*Enterobacter*	Not Detected	42°C W/O Zn	ON845390
177_2_37_1	21-Jun-2021	A3	*Enterobacter*	*bla* $_{IMP-1}$	37°C W/O Zn	ON845391
178_2_37_2	21-Jun-2021	A3	*Aeromonas*		37°C W/O Zn	ON845392
179_2_37_3	21-Jun-2021	A3	*Aeromonas*		37°C W/O Zn	ON845393
180_2_37_4	21-Jun-2021	A3	*Aeromonas*		37°C W/O Zn	ON845394
181_2_37_5	21-Jun-2021	A3	*Aeromonas*		37°C W/O Zn	ON845395
182_2_37_7	21-Jun-2021	A3	*Aeromonas*		37°C W/O Zn	ON845396
183_2_37_9	21-Jun-2021	A3	*Aeromonas*		37°C W/O Zn	ON845397
184_2_37_10	21-Jun-2021	A3	*Aeromonas*		37°C W/O Zn	ON845398
185_2_37_12	21-Jun-2021	A3	*Raoultella*	*bla* $_{GES}$ (CP)	37°C W/O Zn	ON845399
186_2_37_13	21-Jun-2021	A3	*Aeromonas*		37°C W/O Zn	ON845400
187_2_37_16	21-Jun-2021	A3	*Stenotrophomonas*		37°C W/O Zn	ON845401
188_2_37_17	21-Jun-2021	A3	*Enterobacter*	*bla* $_{IMP-1}$	37°C W/O Zn	ON845402
189_2_37_19	21-Jun-2021	A3	*Aeromonas*		37°C W/O Zn	ON845403
190_2_42_1	21-Jun-2021	A3	*Klebsiella*	*bla* $_{GES}$ (CP)	42°C W/O Zn	ON845404
191_a_42_2	12-Jul-2021	A4	*Citrobacter*	*bla* $_{IMP-1}$	42°C W/O Zn	ON845405
192_a_42_10	12-Jul-2021	A4	*Klebsiella*	*bla* $_{IMP-1}$	42°C W/O Zn	ON845406
193_b_37_2	12-Jul-2021	A4	*Stenotrophomonas*		37°C W/O Zn	ON845407
194_b_37_10	12-Jul-2021	A4	*Stenotrophomonas*		37°C W/O Zn	ON845408
195_b_37_11	12-Jul-2021	A4	*Stenotrophomonas*		37°C W/O Zn	ON845409

196_b_37_15	12-Jul-2021	A4	*Enterobacter*	Not Detected	37°C W/O Zn	ON845410
197_a_37_1	28-Jul-2021	A3	*Klebsiella*	bla_{GES} (CP)	37°C W/O Zn	ON845411
198_a_37_4	28-Jul-2021	A3	*Pseudomonas****		37°C W/O Zn	ON845412
199_a_37_6	28-Jul-2021	A3	*Raoultella*	bla_{GES} (CP)	37°C W/O Zn	ON845413
200_a_42_4	28-Jul-2021	A3	*Klebsiella*	bla_{IMP-6}	42°C W/O Zn	ON845414
201_b_37_1	28-Jul-2021	A3	*Enterobacter*	bla_{GES} (CP)	37°C W/O Zn	ON845415
202_b_37_2	28-Jul-2021	A3	*Klebsiella*	Not Detected	37°C W/O Zn	ON845416
203_b_42_1	28-Jul-2021	A3	*Enterobacter*	bla_{GES} (CP) + bla_{IMP-6}	42°C W/O Zn	ON845417
204_b_42_4	28-Jul-2021	A3	*Enterobacter*	Not Detected	42°C W/O Zn	ON845418
205_b_42_5	28-Jul-2021	A3	*Enterobacter*	bla_{IMP-1}	42°C W/O Zn	ON845419
206_b_42_6	28-Jul-2021	A3	*Enterobacter*	Not Detected	42°C W/O Zn	ON845420
207_b_42_7	28-Jul-2021	A3	*Enterobacter*	bla_{IMP-6}	42°C W/O Zn	ON845421
208_b_42_8	28-Jul-2021	A3	*Enterobacter*	Not Detected	42°C W/O Zn	ON845422
209_37_m_3	18-Oct-2021	A3	*Aeromonas*		37°C W/O Zn	ON845423
210_37_m_10	18-Oct-2021	A3	*Aeromonas*		37°C W/O Zn	ON845424
211_37_m_12	18-Oct-2021	A3	*Aeromonas*		37°C W/O Zn	ON845425
212_37_m_15	18-Oct-2021	A3	*Aeromonas*		37°C W/O Zn	ON845426
213_37_z_1	18-Oct-2021	A3	*Klebsiella*	bla_{IMP-6}	37°C with Zn	ON845427
214_37_z_3	18-Oct-2021	A3	*Klebsiella*	bla_{IMP-6}	37°C with Zn	ON845428
215_42_z_1	18-Oct-2021	A3	*Klebsiella*	bla_{IMP-6}	42°C with Zn	ON845429
216_42_z_2	18-Oct-2021	A3	*Klebsiella*	bla_{GES} (CP) + bla_{IMP-6}	42°C with Zn	ON845430
217_42_z_5	18-Oct-2021	A3	*Klebsiella*	bla_{IMP-6}	42°C with Zn	ON845431
218_42_z_7	18-Oct-2021	A3	*Klebsiella*	bla_{IMP-6}	42°C with Zn	ON845432
219_42_z_8	18-Oct-2021	A3	*Citrobacter*	bla_{IMP-6}	42°C with Zn	ON845433
220_48_37_m_4	18-Oct-2021	A3	*Pseudomonas****		37°C W/O Zn	ON845434
221_48_37_m_7	18-Oct-2021	A3	*Pseudomonas****		37°C W/O Zn	ON845435
222_48_42_z_1	18-Oct-2021	A3	*Klebsiella*	bla_{GES} (CP) + bla_{IMP-6}	42°C with Zn	ON845436
223_48_42_z_2	18-Oct-2021	A3	*Klebsiella*	bla_{NDM}	42°C with Zn	ON845437
224_h_37_m_1	28-Nov-2021	A1	*Klebsiella*	bla_{GES} (CP)	37°C W/O Zn	ON844998
225_h_37_m_2	28-Nov-2021	A1	*Klebsiella*	bla_{GES} (CP)	37°C W/O Zn	ON844999
226_h_37_z7_3	28-Nov-2021	A1	*Enterobacter*	Not Detected	37°C with Zn	ON845000
227_h_37_z70_3	28-Nov-2021	A1	*Klebsiella*	bla_{GES} (CP)	37°C with Zn	ON845001
228_h_42_z7_6	28-Nov-2021	A1	*Klebsiella*	bla_{GES} (CP)	42°C with Zn	ON845002
229_h_42_z7_8	28-Nov-2021	A1	*Klebsiella*	bla_{GES} (CP)	42°C with Zn	ON845003

230_h_42_z70_5	28-Nov-2021	A1	*Klebsiella*	*bla* GES (CP)	42°C with Zn	ON845004
231_t2_42_z70_1	6-Dec-2021	A4	*Klebsiella*	Not Detected	42°C with Zn	ON845005
232_t1_37_m_10	9-Dec-2021	A3	*Aeromonas*		37°C W/O Zn	ON845006
233_t1_37_m_12	9-Dec-2021	A3	*Aeromonas*		37°C W/O Zn	ON845007
234_t1_37_m_14	9-Dec-2021	A3	*Aeromonas*		37°C W/O Zn	ON845008
235_t1_37_m_22	9-Dec-2021	A3	*Stenotrophomonas*		37°C W/O Zn	ON845009
236_t1_37_z7_1	9-Dec-2021	A3	*Klebsiella*	*bla* GES (CP) + *bla* IMP-6	37°C with Zn	ON845010
237_t1_37_z7_3	9-Dec-2021	A3	*Klebsiella*	*bla* GES (CP) + *bla* IMP-6	37°C with Zn	ON845011
238_t1_37_z7_5	9-Dec-2021	A3	*Citrobacter*	*bla* GES (CP)	37°C with Zn	ON845012
239_t1_37_z7_9	9-Dec-2021	A3	*Stenotrophomonas*		37°C with Zn	ON845013
240_t1_37_z7_10	9-Dec-2021	A3	*Stenotrophomonas*		37°C with Zn	ON845014
241_t1_37_z7_11	9-Dec-2021	A3	*Stenotrophomonas*		37°C with Zn	ON845015
242_t1_37_z7_12	9-Dec-2021	A3	*Aeromonas*		37°C with Zn	ON845016
243_t1_42_z7_1	9-Dec-2021	A3	*Kluyvera*	*bla* GES (CP)	42°C with Zn	ON845017
244_t1_42_z7_2	9-Dec-2021	A3	*Enterobacter*	*bla* IMP-6	42°C with Zn	ON845018
245_t1_42_z7_3	9-Dec-2021	A3	*Klebsiella*	*bla* GES (CP) + *bla* IMP-6	42°C with Zn	ON845019
246_t1_42_z70_1	9-Dec-2021	A3	*Enterobacter*	*bla* IMP-6	42°C with Zn	ON845020
247_t1_42_z70_2	9-Dec-2021	A3	*Enterobacter*	*bla* IMP-6	42°C with Zn	ON845021

* Treated wastewater taken at a treatment plant (municipal wastewater) located near the mouth of the Tama River.

** Specie was identified based on a part of sequencing results because of the low quality of the sequencing results.

*** The obtained 16S rRNA sequences were close to that of *Stenotrophomonas* sp., although the isolates were tentatively identified as *Pseudomonas* sp.
